# Early intervention with tirzepatide or semaglutide influences anti-atherosclerotic effects in ApoE knockout mice

**DOI:** 10.1038/s41598-026-42437-8

**Published:** 2026-04-07

**Authors:** Kazunori Dan, Junpei Sanada, Tomohiko Kimura, Yuichiro Iwamoto, Toshitomo Sugisaki, Hideyuki Iwamoto, Yoshiro Fushimi, Yuka Nogami, Yoshiko Shirakiya, Masashi Shimoda, Shuhei Nakanishi, Kohei Kaku, Hideaki Kaneto

**Affiliations:** Department of Diabetes, Endocrinology and Metabolism, Kawasaki Medical School, 577 Matsushima, Kurashiki, 701-0192 Japan

**Keywords:** Tirzepatide, Semaglutide, Type 2 diabetes mellitus, Atherosclerosis, Biochemistry, Cardiology, Diseases, Endocrinology, Medical research, Physiology

## Abstract

This study aimed to investigate the anti-atherosclerotic properties of tirzepatide, a dual glucose-dependent insulinotropic polypeptide (GIP)/glucagon-like peptide-1 (GLP-1) receptor agonist, in comparison with semaglutide, a selective GLP-1 receptor agonist. ApoE knockout mice were divided into early diabetes (dosed from 10 to 22 weeks of age), late diabetes (dosed from 18 to 30 weeks of age), and non-diabetic groups after streptozotocin treatment, and each group received semaglutide, tirzepatide, or saline for 12 weeks. In the early diabetes group, both agents significantly suppressed aortic plaque formation compared with control, while modestly improving glycemia and lipid levels. No significant vascular effects were observed in late diabetes or non-diabetic groups. Tirzepatide markedly reduced inflammatory mediators, including *Mcp-1*, *Il-6*, *I-cam*, and *Cd68*, whereas semaglutide showed partial overlap. Notably, these anti-inflammatory effects were also detected in non-diabetic mice, suggesting vascular protection may involve arterial actions independently of metabolic control. Taken together, our findings demonstrate that tirzepatide exerts anti-atherosclerotic effects comparable to semaglutide, supporting the concept that GIP and GLP-1 signaling can confer vascular benefits. These results highlight the potential clinical relevance of dual incretin receptor agonism for cardiovascular risk reduction, although further studies are required to clarify the specific role of GIP signaling.

**Supplementary Information:**

The online version contains supplementary material available at 10.1038/s41598-026-42437-8.

## Introduction

Atherosclerosis is a major pathological condition involved in the development of many diseases, including cardiovascular disease, stroke, and peripheral arterial disease^1^. Especially in patients with type 2 diabetes mellitus (DM), chronic inflammation, oxidative stress, and abnormal lipid metabolism associated with hyperglycemia are known to accelerate atherosclerosis and increase the risk of cardiovascular events^2–4^. Against this background, the establishment of therapeutic strategies to reduce the progression of atherosclerosis in diabetic patients is an extremely important clinical issue. Recently, it has been suggested that glucagon-like peptide-1 receptor agonists (GLP-1RAs) are protective against the development of atherosclerosis. Large scale clinical trials such as the LEADER trial, SUSTAIN-6 trial, Harmony Outcomes, PIONEER trial, REWIND trials have demonstrated the cardiovascular benefits of GLP-1RAs^5–9^. We reported that expression level of GLP-1R in endothelial and smooth muscle cells in blood vessels was significantly reduced in obese type 2 diabetic *db/db* mice compared to non-diabetic mice^10^. Additionally, we showed the first report demonstrating that GLP-1R was present in human blood vessels^11^. These findings strongly support the hypothesis that GLP-1RAs exert vascular protective effects, which may contribute to the improvement of major adverse cardiovascular events observed with GLP-1RA treatment in addition to their effects through metabolic improvement. Previous studies have shown that GLP-1RAs may contribute to the stabilization of atherosclerotic plaques through the promotion of nitric oxide (NO) production, anti-inflammatory effects, inhibition of macrophage activity, reduction of oxidative stress, and improvement of lipid metabolism^12–20^. Furthermore, although glucose-dependent insulinotropic polypeptide (GIP) receptor agonist (GIP-RA) has not been studied as extensively as GLP-1RAs, it was clearly demonstrated that administration of GIP-RA enhanced insulin sensitivity^21^. Furthermore, it has been reported that administration of human GIP or overexpression of GIP suppresses atherosclerosis^22,23^. Additionally, it has been suggested that knockdown of GIP-R promotes atherosclerosis^24^. In particular, it has been pointed out that tirzepatide, a GIP/GLP-1RA, may have potent anti-atherosclerosis effects through its actions on immune cells such as adipocytes and macrophages^25,26^. However, how tirzepatide affects the development of atherosclerosis and the detailed mechanism of its action remained largely unexplored.

In this study, we evaluated the development of atherosclerotic lesions in the aorta of mice treated with semaglutide or tirzepatide. Then, we compared the possible effect of tirzepatide with established effect of semaglutide on the development of atherosclerosis and clarify its possible underlying mechanism.

## Results

### Semaglutide and tirzepatide improved glycemic control in STZ-induced hyperglycemic mice

**Fig. 1:**
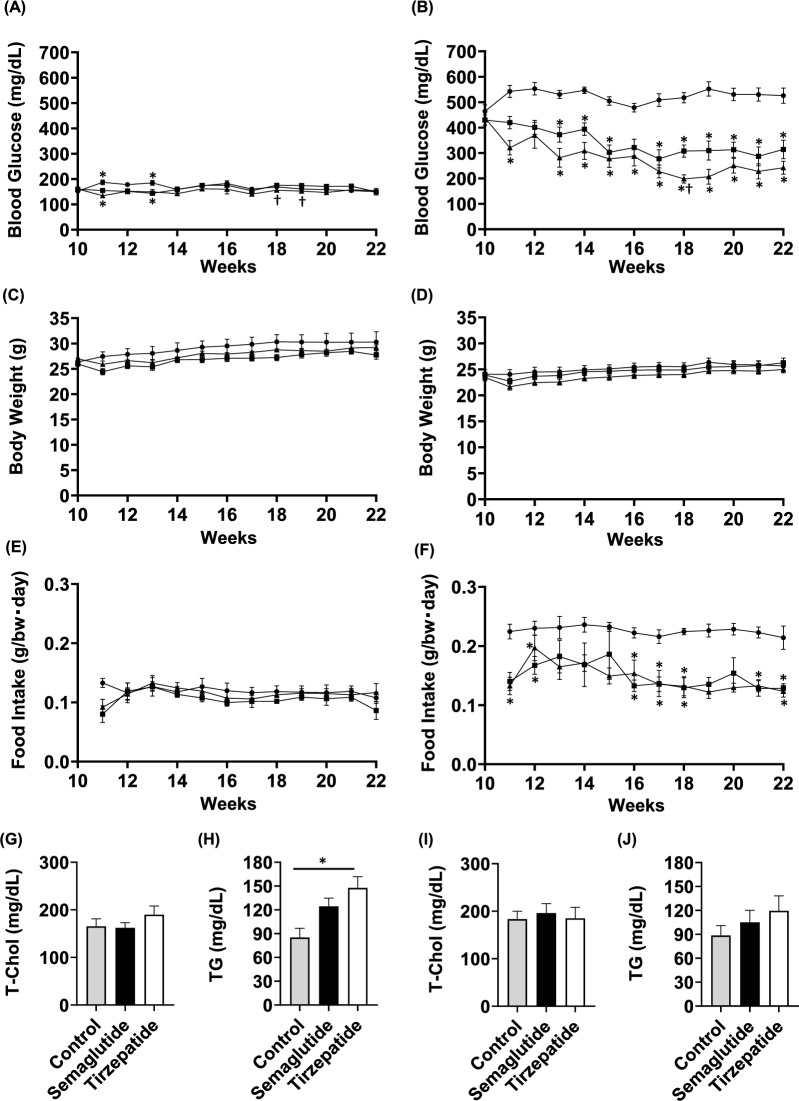
Plaque formation in the aortic arch was significantly reduced by semaglutide or tirzepatide treatment in early DM group. (**A**–**H**) Blood glucose levels, body weight, and serum lipid concentration in early intervention ApoE knockout mice when semaglutide (800 μg/kg × 2/week), tirzepatide (150 μg/kg × 2/week) or vehicle was subcutaneously administered. (**A**,**C**) Blood glucose levels and body weight in the early non-DM group (**B**,**D**) Blood glucose levels and body weight in the early DM group. (**E**) Food intake levels in the early non-DM group. (**F**) Food intake levels in the early DM group. (**G**,**H**) Serum T-Chol and serum TG after intervention in the early non-DM group. (**I**,**J**) Serum T-Chol and serum TG after intervention in the early DM group. Black circle and bars, control group; Black squares and bars, semaglutide group; Black triangle and bars, tirzepatide group. n = 8–11 (early non-DM group), n = 13–18 (early DM group). *p < 0.05 (control vs semaglutide, control vs tirzepatide), †p < 0.05 (semaglutide vs tirzepatide).

In non-DM ApoE knockout mice (early non-DM group) treated with the drugs from 10 to 22 weeks of age, semaglutide and tirzepatide administration did not significantly affect blood glucose levels (Fig. 1A). However, in STZ-induced hyperglycemic mice (early DM group) treated with the drugs for the same period, glycemic control was significantly improved in the semaglutide and tirzepatide groups (Fig. 1B). Body weight was comparable among the control, semaglutide, and tirzepatide groups in these models (Fig. 1C,D). For food intake, there was no significant difference in the early non-DM group except at baseline (Fig. 1E), however, in the early DM group, food intake was lower in the semaglutide group and the tirzepatide group compared to the control group (Fig. 1F). Regarding lipid profiles, in the early non-DM group, there was no difference in total cholesterol levels (Fig. 1G). Triglyceride levels were higher in the tirzepatide group than control group (Fig. 1H). In the early DM group, there was no difference in total cholesterol and triglyceride levels among the three groups (Fig. 1I,J). Furthermore, in the early DM group, neither the high-performance liquid chromatography (HPLC)-derived elution profiles of plasma lipoprotein fractions (Supplemental Fig. 1A–C) nor the quantitative distribution of each fraction (Supplemental Table 1) showed significant differences among the groups. No differences were observed in systolic and diastolic blood pressure and heart rate among the control, semaglutide, and tirzepatide groups (Tables 1 and 2A). Regarding organ weight, in the early non-DM group, the white adipose tissue (WAT) weight was smaller in the semaglutide group compared to the control group, but no significant differences were observed among the groups for other organs (Table 1). In the early DM group, no significant differences were observed among the groups for any organ (Table 2A).

**Table 1:** Blood pressure, heart rate and organ weights in early non-DM group.

	Before treatment (10 weeks)			After treatment (22 weeks)			
	Control	Semaglutide	Tirzepatide	Control	Semaglutide	Tirzepatide	
Systolic BP (mmHg)	102.7 ± 5.0	107.1 ± 4.6	109.0 ± 3.1	107.3 ± 3.6	111.5 ± 5.8	104.1 ± 6.7	
Diastolic BP (mmHg)	47.2 ± 6.5	54.1 ± 3.5	54.0 ± 7.3	43.4 ± 5.4	52.5 ± 4.2	49.5 ± 8.0	
Heart rate (beats/min)	675.0 ± 14.6	702.5 ± 12.0	698.9 ± 10.7	675.8 ± 9.3	688.8 ± 12.0	697.9 ± 11.6	
Heart (g)	–	–	–	0.133 ± 0.011	0.125 ± 0.006	0.122 ± 0.006	
Kidney (g)	–	–	–	0.339 ± 0.031	0.346 ± 0.006	0.360 ± 0.013	
Liver (g)	–	–	–	1.089 ± 0.095	1.047 ± 0.034	1.156 ± 0.055	
Pancreas (g)	–	–	–	0.226 ± 0.026	0.283 ± 0.013	0.254 ± 0.007	
Muscle (g)	–	–	–	0.366 ± 0.022	0.337 ± 0.007	0.343 ± 0.009	
White adipose tissue (g)	–	–	–	0.372 ± 0.076	0.185 ± 0.023*	0.207 ± 0.021	
Brown adipose tissue (g)	–	–	–	0.107 ± 0.010	0.109 ± 0.007	0.113 ± 0.008	

*p < 0.05 ( vs. Con).

**Table 2:** (A) Blood pressure, heart rate and organ weights in early DM group, (B) Blood pressure, heart rate and organ weights in late DM group.

(A)						
	Before treatment (10 weeks)			After treatment (22 weeks)		
	Control	Semaglutide	Tirzepatide	Control	Semaglutide	Tirzepatide
Systolic BP (mmHg)	110.3 ± 4.4	119.9 ± 6.2	115.0 ± 4.9	102.9 ± 3.2	115.7 ± 6.1	110.7 ± 4.2
Diastolic BP (mmHg)	55.8 ± 6.4	58.3 ± 8.6	58.2 ± 5.1	58.0 ± 5.3	59.8 ± 8.9	58.4 ± 4.2
Heart rate (beats/min)	645.2 ± 16.7	680.5 ± 11.9	673.0 ± 11.4	681.8 ± 15.7	660.9 ± 9.3	660.9 ± 11.6
Heart (g)	–	–	–	0.102 ± 0.005	0.096 ± 0.008	0.092 ± 0.005
Kidney (g)	–	–	–	0.328 ± 0.009	0.346 ± 0.050	0.296 ± 0.013
Liver (g)	–	–	–	1.076 ± 0.041	0.986 ± 0.044	0.979 ± 0.030
Pancreas (g)	–	–	–	0.224 ± 0.013	0.230 ± 0.021	0.231 ± 0.017
Muscle (g)	–	–	–	0.286 ± 0.016	0.268 ± 0.035	0.269 ± 0.018
White adipose tissue (g)	–	–	–	0.059 ± 0.030	0.053 ± 0.016	0.052 ± 0.010
Brown adipose tissue (g)	–	–	–	0.092 ± 0.009	0.098 ± 0.014	0.093 ± 0.008

(B)						
	Before treatment (18 weeks)			After treatment (30 weeks)		
	Control	Semaglutide	Tirzepatide	Control	Semaglutide	Tirzepatide
Systolic BP (mmHg)	109.2 ± 5.1	110.7 ± 6.4	101.0 ± 4.3	105.8 ± 3.6	112.0 ± 6.7	116.2 ± 8.9
Diastolic BP (mmHg)	57.2 ± 4.9	60.7 ± 7.3	49.8 ± 3.0	56.1 ± 4.3	50.1 ± 5.1	67.8 ± 7.7^†^
Heart rate (beats/min)	699.5 ± 14.5	706.9 ± 14.1	698.1 ± 19.5	663.2 ± 9.6^†^	666.8 ± 13.8	673.9 ± 14.3
Heart (g)	–	–	–	0.118 ± 0.005	0.132 ± 0.008	0.113 ± 0.006
Kidney (g)	–	–	–	0.385 ± 0.014	0.398 ± 0.019	0.363 ± 0.026
Liver (g)	–	–	–	1.463 ± 0.089	1.266 ± 0.081	1.207 ± 0.086
Pancreas (g)	–	–	–	0.243 ± 0.011	0.265 ± 0.016	0.250 ± 0.013
Muscle (g)	–	–	–	0.326 ± 0.017	0.300 ± 0.022	0.291 ± 0.019
White adipose tissue (g)	–	–	–	0.094 ± 0.023	0.068 ± 0.021	0.107 ± 0.027
Brown adipose tissue (g)	–	–	–	0.106 ± 0.010	0.096 ± 0.006	0.102 ± 0.006

^†^p < 0.05 (18 vs 30 weeks within the same group).

### Plaque formation in the aortic arch was significantly reduced by semaglutide or tirzepatide treatment in early DM group

**Fig. 2:**
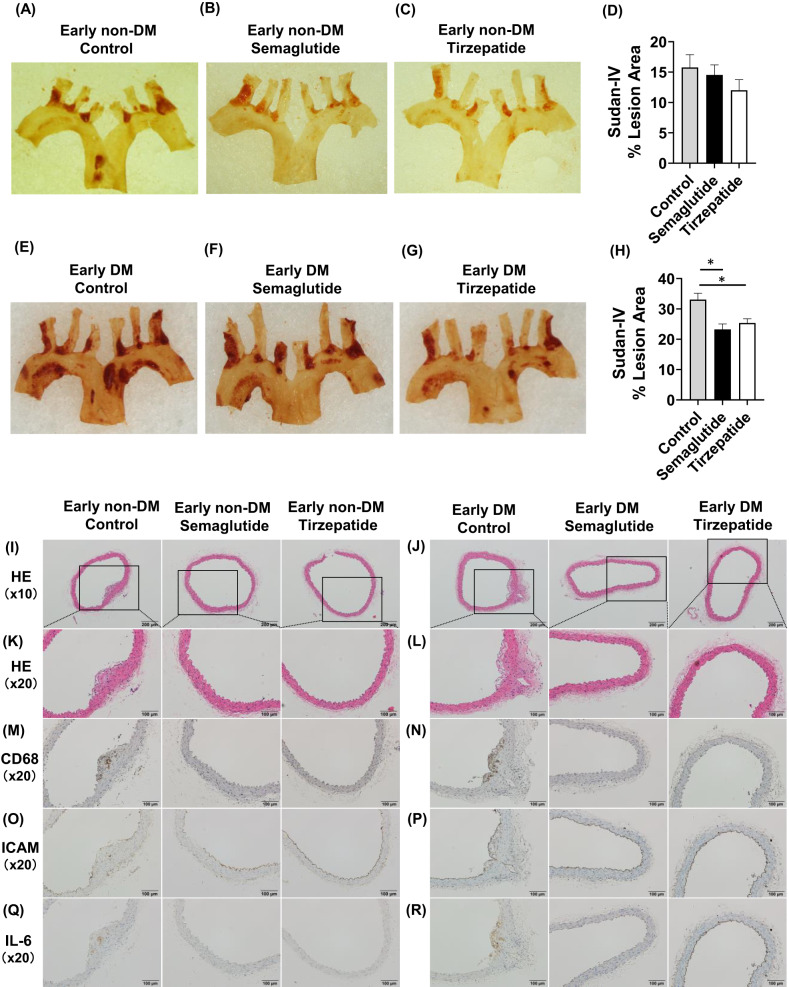
(**A**–**D**) Atherosclerotic lesions in the aortic arch of ApoE knockout mice. (**A**–**C**) Representative Sudan IV staining of the aortic arch in the early non-DM group. (**D**) Sudan IV positive areas (% of lesion area) in the early non-DM group. (**E**–**G**) Representative Sudan IV staining of aortic arch in early DM group. (**H**) Sudan IV-positive areas (% of lesion area) in the early DM group. (**I**,**J**) Representative HE staining of the thoracic aorta at 10 × magnification in the early non-DM and early DM groups. (**K**,**L**) Representative HE staining of the thoracic aorta at 20 × magnification in the early non-DM and early DM groups. (**M**,**N**) Representative CD68 staining of the thoracic aorta at 20 × magnification in the early non-DM and early DM groups. (**O**,**P**) Representative I-CAM staining of the thoracic aorta at 20 × magnification in the early non-DM and early DM groups. (**Q**,**R**) Representative IL-6 staining of the thoracic aorta at 20 × magnification in the early non-DM and early DM groups. n = 8–11 (early non-DM group), n = 13–18 (early DM group). *p < 0.05.

Next, we evaluated the effects of semaglutide and tirzepatide on the progression of atherosclerosis. No statistically significant difference was observed in Sudan IV staining among the semaglutide, tirzepatide, and control groups in the early non-DM group (Fig. 2A–D). However, in the early DM group, administration of semaglutide and tirzepatide significantly suppressed the progression of plaque formation in the aortic arch compared to the control group. No significant difference was observed between the semaglutide and tirzepatide group (Fig. 2E–H). These data suggest that semaglutide and tirzepatide exert anti-atherosclerotic effects to the similar extent in diabetic mice.

### In atherosclerotic lesions, infiltration of macrophages was observed, but it was significantly suppressed by semaglutide or tirzepatide treatment

To investigate plaque characteristics, hematoxylin and eosin (HE) staining and immunohistochemistry were performed on the thoracic aorta of early non-DM group and early DM group. In the control group, HE staining revealed prominent plaque formation. However, mice administered semaglutide or tirzepatide showed markedly attenuated plaque development compared with controls (Fig. 2I–L). To evaluate macrophage infiltration into the vascular wall, CD68 staining was conducted. In the control group, extensive CD68-positive areas were observed, corresponding to the plaque regions. However, mice treated with semaglutide or tirzepatide exhibited a pronounced reduction in CD68-positive areas relative to controls (Fig. 2M,N). To assess endothelial inflammatory activation and leukocyte recruitment, I-CAM staining was conducted. In the control group, I-CAM-positive areas were observed in association with the plaque regions, whereas mice administered semaglutide or tirzepatide showed fewer I-CAM-positive areas relative to controls (Fig. 2O,P). Furthermore, immunohistochemical staining for IL-6 was performed to evaluate the expression of inflammatory cytokines. While the control group exhibited intense IL-6-positive staining within the plaque regions, this staining was slightly attenuated in the groups treated with semaglutide or tirzepatide (Fig. 2Q,R).

### Semaglutide and tirzepatide also improved glycemic control in late DM group

**Fig. 3:**
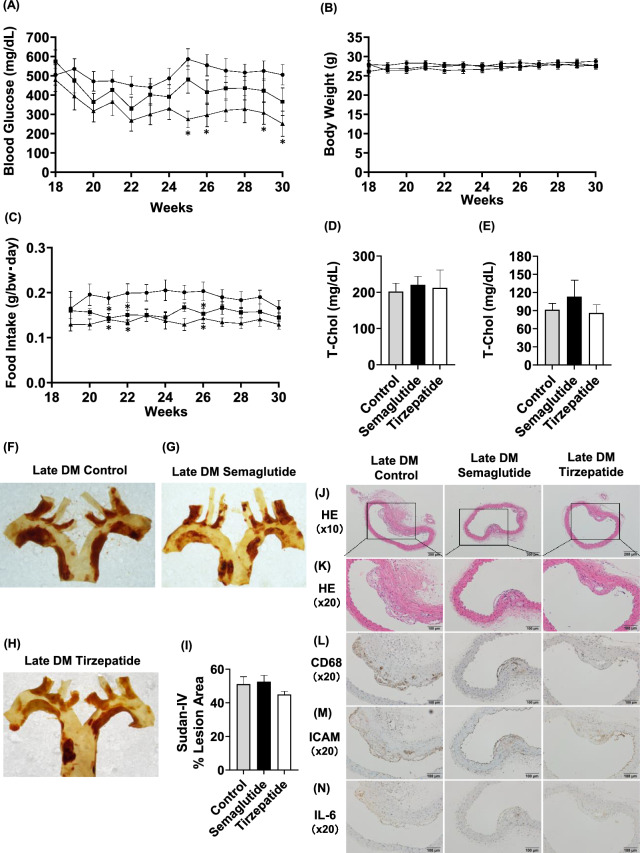
In the late DM group, plaque formation in the aortic arch was not changed by semaglutide or tirzepatide treatment. (**A**–**E**) Blood glucose levels, body weight, and food intake, serum lipid concentration in late intervention ApoE knockout mice when semaglutide, tirzepatide or vehicle was subcutaneously administered. (**A**) Blood glucose levels, (**B**) body weight, (**C**) food intake, (**D**,**E**) serum T-Chol, serum TG after intervention (**F**–**I**). Atherosclerotic lesions of the aortic arch in the late intervention group of ApoE knockout mice. (**F**–**H**) Representative Sudan IV staining of aortic arch. (**I**) Sudan IV positive area (% of lesion area). (**J**) Representative HE staining of the thoracic aorta at 10 × magnification. (**K**) Representative HE staining of the thoracic aorta at 20 × magnification. (**L**) Representative CD68 staining of the thoracic aorta at 20 × magnification. (**M**) Representative I-CAM staining of the thoracic aorta at 20 × magnification. (**N**) Representative IL-6 staining of the thoracic aorta at 20 × magnification. Black circle and bars, control group; Black squares and bars, semaglutide group; Black triangle and bars, tirzepatide group. n = 9–12 (late DM group). *p < 0.05 (control vs semaglutide, control vs tirzepatide), ^†^p < 0.05 (semaglutide vs tirzepatide).

We evaluated data from hyperglycemic ApoE knockout mice treated with the drugs from 18 to 30 weeks of age (late DM group). Glycemic control was significantly improved in the tirzepatide group compared with the control group (Fig. 3A). Body weight was similar in the control, semaglutide, and tirzepatide groups (Fig. 3B). Food intake was similarly reduced in the semaglutide group as in the tirzepatide group compared to the control group (Fig. 3C).

Similarly, lipid profile (total cholesterol and triglycerides) did not differ among the three groups (Fig. 3D,E). Furthermore, blood pressure, pulse rate, and organ weights did not differ significantly among the three groups (Table 2B).

### In the late DM group, plaque formation in the aortic arch was not changed by semaglutide or tirzepatide treatment

Next, to examine the effects of semaglutide and tirzepatide on the progression of atherosclerosis in the late DM group, we evaluated plaque formation in the aortic arch at 30 weeks of age. The results showed no statistically significant difference in Sudan IV staining in either group (Fig. 3F–I). Histological analysis revealed evident plaque formation in the control group as assessed by HE staining, and, in contrast to the early non-DM and early DM groups, comparable plaque formation was also observed in the semaglutide- and tirzepatide-treated groups (Fig. 3J,K). Furthermore, plaque-associated CD68-positive macrophage areas (Fig. 3L), I-CAM-positive areas (Fig. 3M), and IL-6–positive areas (Fig. 3N) were not improved by treatment in the late DM group.

### Semaglutide and tirzepatide were associated with reduced expression of inflammatory markers, cell adhesion factors, and macrophage-related genes

**Fig. 4:**
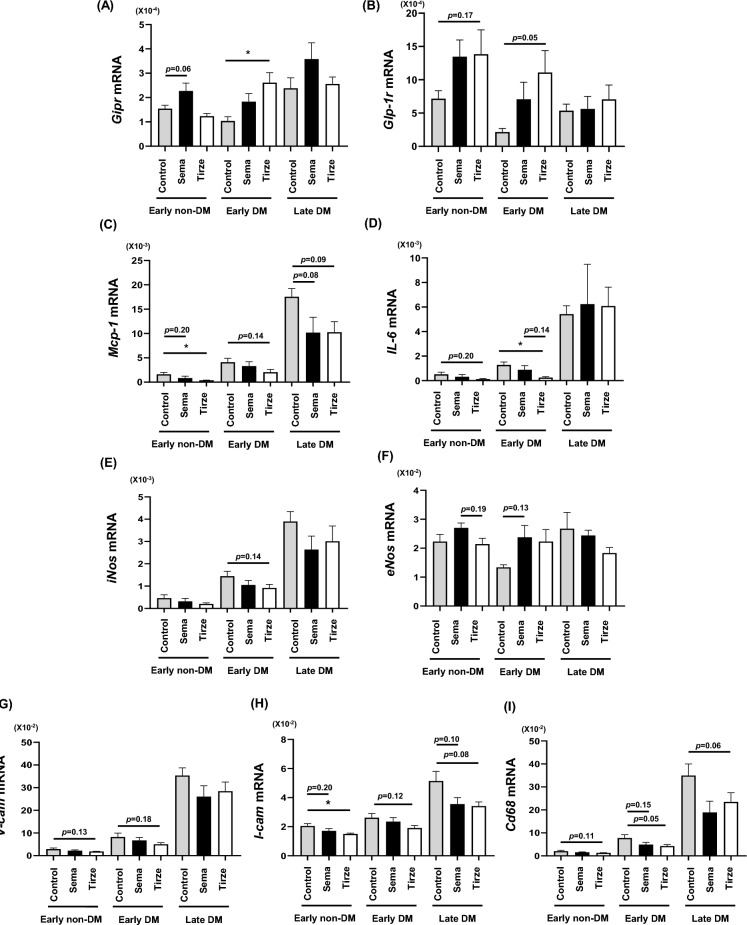
mRNA expression levels of incretin receptor, and nitric oxide synthase, and inflammation-, coagulation-, cell adhesion- and macrophage-related markers in the abdominal aorta. (**A**,**B**) mRNA expression levels of *GIPR* and *GLP-1R*. (**C**,**D**) mRNA expression levels of inflammation markers such as *Mcp-1* and *IL-6*. (E,**F**) mRNA expression levels of nitric oxide synthase such as i*Nos* and *eNos*. (**G**,**H**) mRNA expression levels of cell adhesion factors such as *V-cam* and *I-cam*. (**I**) mRNA expression levels of macrophage-related gene *Cd68*. n = 7–8 (early non-DM group), n = 12–15 (early DM group), n = 7–9 (late DM group). *p < 0.05.

To further examine the protective effects of semaglutide and tirzepatide against atherosclerosis, we evaluated the mRNA expression levels of incretin receptor, NO-producing enzymes, inflammatory markers, cell adhesion factors and macrophage-related genes in the abdominal aorta. mRNA expression levels of multiple atherosclerosis-related genes, excluding *Gipr*, *Glp-1r* and *eNos* were higher in the late intervention group (late DM group) compared to the early intervention group (early non-DM group, early DM group) (Fig. 4A,B,F). In the early DM group, *Gipr* mRNA expression levels were higher in the tirzepatide group compared to the control group (Fig. 4A). *Mcp-1* levels were significantly lower in the tirzepatide group in the early non-DM group; however, no statistically significant differences were observed among the control, semaglutide, and tirzepatide groups in the early DM and late DM groups (Fig. 4C). *IL-6* levels were not significantly different in the early non-DM group, whereas they were significantly lower in the tirzepatide group in the early DM group (Fig. 4D). Expression levels of *iNos* showed no significant differences among the control, semaglutide and tirzepatide groups in the early non-DM, early DM, and late DM groups (Fig. 4E). *eNos* levels were numerically higher in the semaglutide group in the early DM group (Fig. 4F). For *V-cam* levels, no statistically significant differences were observed among the groups in the early non-DM and early DM groups (Fig. 4G). Similarly, *I-cam* expression levels did not differ significantly among the groups across all three models (Fig. 4H). Regarding macrophage-related genes, *Cd68* expression levels did not show statistically significant differences among the groups; mean values were numerically lower in the semaglutide and tirzepatide groups compared to the control group across all three models (Fig. 4I).

## Discussion

This study demonstrated that the incretin receptor agonists semaglutide and tirzepatide exhibited anti-atherosclerotic effects in a STZ-induced diabetic ApoE knockout mouse model under conditions. It is noted here again that the drug dosages were adjusted to achieve comparable blood glucose levels in the non-DM models, so that we could minimize the effects of difference in blood glucose levels on the results.

In the present study, diabetic models treated with semaglutide or tirzepatide in the early intervention group significantly reduced plaque formation in the aortic arch. However, semaglutide and tirzepatide did not reduce atherosclerotic plaque in non-DM models and in late intervention groups. One possible reason is that atherosclerosis was relatively mild in untreated non-DM model compared to untreated DM model and thus the difference between the medication and control groups was relatively small. On the other hand, in the late intervention group, the duration of exposure to hyperglycemia was longer and plaque had already been formed extensively, which may have reduced the protective effect of the medication against plaque formation. This indicates that once atherosclerosis has been formed, it is difficult to inhibit its progression, indicating the importance of early glycemic and inflammatory control.

It is also possible that the effect of these drugs may have been limited because previous studies have reported that GLP-1 and GIP receptors are downregulated by glucose toxicity^27^. Furthermore, unlike some previous reports^21^, no weight loss was observed in models treated with semaglutide or tirzepatide in this study. While many previous reports^21^ used mouse models with diet-induced obesity, this study employed non-obese mouse models with reduced insulin secretion capacity induced by streptozotocin administration. Therefore, the lower body weight of the model mice at the time of intervention likely resulted in a relatively smaller degree of weight reduction upon administration of semaglutide or tirzepatide compared to previous reports. However, in the early non-DM group, early DM group, and late DM group, administration of semaglutide or tirzepatide affected the expression of atherosclerosis-related factors. In previous studies, GLP-1RA reduced the mRNA expression of inflammatory markers, cell adhesion molecules, and macrophage-related genes^14,28,29^. In addition, administration of tirzepatide to C57BL/6 mice suppressed the expression levels of inflammatory cytokines such as *TNF-α*, *IL-6*, and *IL-1β*^30^. In addition, administration of GIP-RA and GLP-1RA to APOE*3-Leiden.CETP mice reduced the expression levels of monocyte- and macrophage-related factors such as *F4/80*, *Cd68*, and *Mcp-1*, as well as the cell adhesion molecules *I-cam-1* and *V-cam-1*^31^. In the gene expression analysis, treatment effects were marker- and group-specific. A significant reduction was observed for *Mcp-1* and *I-cam* expression levels only in the early non-diabetic tirzepatide group, whereas *Il-6* and *Cd68* levels did not show statistically significant differences across the treatment groups. In diabetic models, the change in atherosclerosis-related factor expression levels might be attributed to the blood glucose-lowering effect. However, expression levels of some genes were altered by semaglutide or tirzepatide in non-diabetic models. These data suggest the possibility that GIP signaling, as well as GLP-1 signaling, has a protective effect on blood vessels.

The present study focused on semaglutide and tirzepatide as incretin receptor agonists, but these agents have different properties. Semaglutide is a pure GLP-1RA, whereas tirzepatide is known to have a high affinity for the GIP-R^32^. While similarities exist in the physiological effects of GLP-1 and GIP signaling, several distinctions are also observed. For instance, adipocytes lack the expression of GLP-1R, whereas GIP-R is abundantly expressed in these cells, enabling GIP to exert direct effects on adipose tissue^33^.

Although GLP-1RA have been shown to inhibit atherosclerosis in clinical trials such as the previous LEADER study, it is possible that tirzepatide, which has a stronger effect on GIP-R, may exert its anti-atherosclerotic effect through a different mechanism of action than GLP-1RA. The inhibitory effect of GIP-RA on atherosclerosis has attracted much attention in recent basic research. Previous studies show that mice lacking the GIP-R show more significant arteriosclerosis progression compared to control mice despite having lower body weight, and their blood vessels exhibit increased expression of inflammation-related genes, suggesting that GIP-R stimulation may directly regulate vascular inflammation. In particular, GIP-R are expressed in macrophages and endothelial cells, and GIP stimulation may improve vascular endothelial function through anti-inflammatory effects and enhancement of NO production^22,34–37^.

The novelty of this study is that it demonstrates comparable anti-atherosclerotic efficacy of tirzepatide, a GIP/GLP-1RA, to GLP-1RA in the STZ-ApoE (-/-) mouse model with early therapeutic intervention. Previous studies focused primarily on the anti-atherosclerotic effects of GLP-1RA, with limited knowledge of the vascular effects of dual GIP/GLP-1RA. This study demonstrates that tirzepatide exerts anti-inflammatory and vasoprotective effects, providing important foundational evidence for its role in future cardiovascular outcome trials. Since this investigation did not directly examine the involvement of GIP or GLP-1 receptor, further studies are necessary to clarify the precise mechanisms of action mediated through these receptors.

Several limitations of this study should be addressed. First, we utilized a non-obese, insulin-deficient diabetic model induced by STZ. Since these mice had low baseline body weight and minimal fat accumulation, the weight-reducing effects typically associated with semaglutide or tirzepatide were not prominently observed. While this model allowed us to evaluate the direct vascular effects independent of weight loss, it may have masked additional anti-atherosclerotic signals that could be mediated by the improvement of obesity-related metabolic dysfunction. Second, the doses of semaglutide and tirzepatide used were high compared to standard clinical doses. Although these doses were determined based on species differences in receptor affinity and our preliminary studies, some caution is required when applying these findings to clinical practice. Next, due to wide, graded staining intensities, reproducible threshold-based quantification of CD68, ICAM-1, and IL-6 was not feasible in this study. Thus, we present representative images and avoid over-interpretation. Quantitative histological validation will be required in future work.

In conclusion, the data in this study suggest that tirzepatide, a GIP/GLP-1RA, has an anti-atherosclerotic effect which is similar to that of semaglutide, a GLP-1RA. These results suggest the possibility that GIP and GLP-1 signaling shows similar favorable effects on the progression of atherosclerosis. The data in this study also demonstrated the importance of early intervention with tirzepatide or semaglutide. Furthermore, this study suggested that these drugs would have great potential for the clinical application as a cardiovascular prevention strategy.

## Methods

### Animals and diets

ApoE knockout mice (C57BL/6 J-ApoEtm1Unc) were purchased from Charles River Laboratories and housed under a 12 h light–dark cycle (2 mice per cage for all experiments) in a controlled environment. Male mice were used to eliminate the confounding effects of the estrous cycle-related hormonal fluctuations on vascular and metabolic parameters. Animals were allowed free access to water and housed at 25 °C. In this experiment, in order to minimize the impact on metabolism due to changes in mouse body weight, the mice were given a standard diet (MF, Oriental Yeast Co., Ltd.) to prevent the induction of obesity. To induce hyperglycemia, STZ (Fujifilm Wako Pure Chemical Industries, Japan) was used based on a previous report^38^. At 8 weeks of age, mice were intraperitoneally administered STZ (50 mg/kg/day) for 5 consecutive days. Diabetes was diagnosed when mice showed obvious hyperglycemia over 300 mg/dL under feeding conditions. Regarding the drug dosage, based on a previous report^39^, we started by subcutaneously injecting the drugs at the same dose twice a week, and adjusted the dosage so that both drugs reduced blood glucose levels to the same extent in the -DM and non-DM model mice used in this study in order to minimize the influence of some difference in blood glucose levels on the results. The titration program initiated the administration of tirzepatide and semaglutide to non-hyperglycemic mice at a ratio of approximately 1:3, based on the previously reported study^21^. The dose of semaglutide was adjusted so that the blood glucose levels in the semaglutide-administered group would be similar to those in the tirzepatide-administered group under the conditions of our laboratory. The administration interval was set to twice weekly, referencing the report by Ma et al^40^. Diabetic ApoE knockout mice were treated with tirzepatide (150 μg/kg, subcutaneous injection twice a week, purchased from Eli Lilly and Company) or semaglutide (800 μg/kg, subcutaneous injection twice a week, purchased from Novo Nordisk Pharma). The control group received phosphate-buffered saline (40–50 μL/subcutaneous injection twice a week). These groups were divided into a model in which drugs were administered from 10 to 22 weeks of age (early DM group) and a model in which drugs were administered from 18 to 30 weeks of age (late DM group).

In order to exclude the influence of blood glucose reduction induced by administration of semaglutide and tirzepatide, the effects of semaglutide and tirzepatide were also investigated in non-diabetic ApoE knockout mice without STZ administration (10–22 weeks of age, early non-diabetic group). Blood glucose levels and body weight were measured during the experimental period. Mice were anesthetized with sevoflurane, induction at 3–4% in air and maintenance at 1.5–2% in air. At the endpoint, mice were euthanized by overdose of sevoflurane. This study was approved by the Kawasaki Medical School Animal Use Committee (Study No. 23-038) and was conducted in accordance with the Kawasaki Medical School Animal Use Guidelines. This study is reported in accordance with the ARRIVE guidelines.

### Measurement of biochemical markers

Blood samples were collected from the tail vein. Random blood glucose levels were measured using a glucometer (Glutest Mint; Sanwa Kagaku Kenkyusho Co, Ltd, Japan). Plasma total cholesterol and triglycerides were measured enzymatically using Wako LabAssay, L type Wako (Wako Pure Chemical Industries, Japan). Lipid profile measurements were performed by LipoSEARCH (Immuno-Biological Laboratories) (Gumma, Japan).

### Blood pressure measurement

Blood pressure and heart rate were measured at the beginning and end of the intervention. Measurements were performed using a computerized non-invasive tail cuff system (NP-NIBP MONITOR FOR MICE & RATS, MUROMACHI KIKAI Co, Ltd, Japan). Animals were placed in mouse holders and blood pressure was measured three times per mouse.

### RNA isolation and real time RT-PCR

Total RNA extraction was performed using a RNeasy lipid tissue mini kit (QIAGEN, Valencia, CA) according to the manufacturers’ instructions. cDNA was produced from mRNA using TaqMan reverse transcription reagents (Applied Biosystems, Foster City, CA). Quantitative RT-PCR was conducted using a Step One Plus Real-Time PCR system (Applied Biosystems). To quantify gene expression, the 2^−ΔCT^ was calculated using β-actin as an internal control.

### Histological and immunohistological analyse

PBS was perfused from the left ventricle under anesthesia, animals were killed and the heart and aorta were dissected. Sudan IV (Wako: 192-04392) staining was performed on the aortic arch. The adventitial adipose tissue was removed and the aorta was dissected longitudinally. Image analysis software NIH Image (version 1.61; http://rsbweb.nih.gov/ij/) was used to calculate the ratio of plaque lesions to total aortic arch area. Isolated thoracic aorta was fixed in formalin at 4 °C. The tissue was processed for paraffin embedding, and 4-μm sections were prepared and mounted on silane-coated slides. Sections were then subjected to HE staining and immunohistochemistry for CD68 (ab125212, RRID:AB_10975465, 1:100), I-CAM1 (ab179707, RRID:AB_2732800, 1:2000), and IL-6 (AF-406-NA, RRID:AB_354477, 1:40).

### Statistical analysis

All data were expressed as mean ± standard error of the mean. Statistical significance among the three groups (control, semaglutide, and tirzepatide) was determined using a one-way analysis of variance (ANOVA), followed by Tukey’s post-hoc test for multiple comparisons. For the statistical evaluation of mRNA expression levels in Fig. 4, a one-way ANOVA followed by Tukey’s post-hoc test was performed to compare the three groups (control, semaglutide, and tirzepatide) within each of the following categories: early non-DM, early DM, and late DM groups. p values less than 0.05 were considered to indicate a statistically significant difference. All data processing and statistical analyses were performed using GraphPad Prism 8 (GraphPad Software, CA, USA).

## Supplementary Information

Supplementary Information 1.

Supplementary Information 2.

## Data Availability

All data supporting the findings of this study are available from the corresponding author upon reasonable request.
