# Supplementary material for: Early intervention with tirzepatide or semaglutide influences anti-atherosclerotic effects in ApoE knockout mice
Source: Sci Rep. 2026 Apr 7;16:16718. doi: 10.1038/s41598-026-42437-8 (PMC13223216; doi:10.1038/s41598-026-42437-8)
Supplement: Supplementary file 1 — Supplementary Information 1. [file 41598_2026_42437_MOESM1_ESM.pptx]

## Slide 1
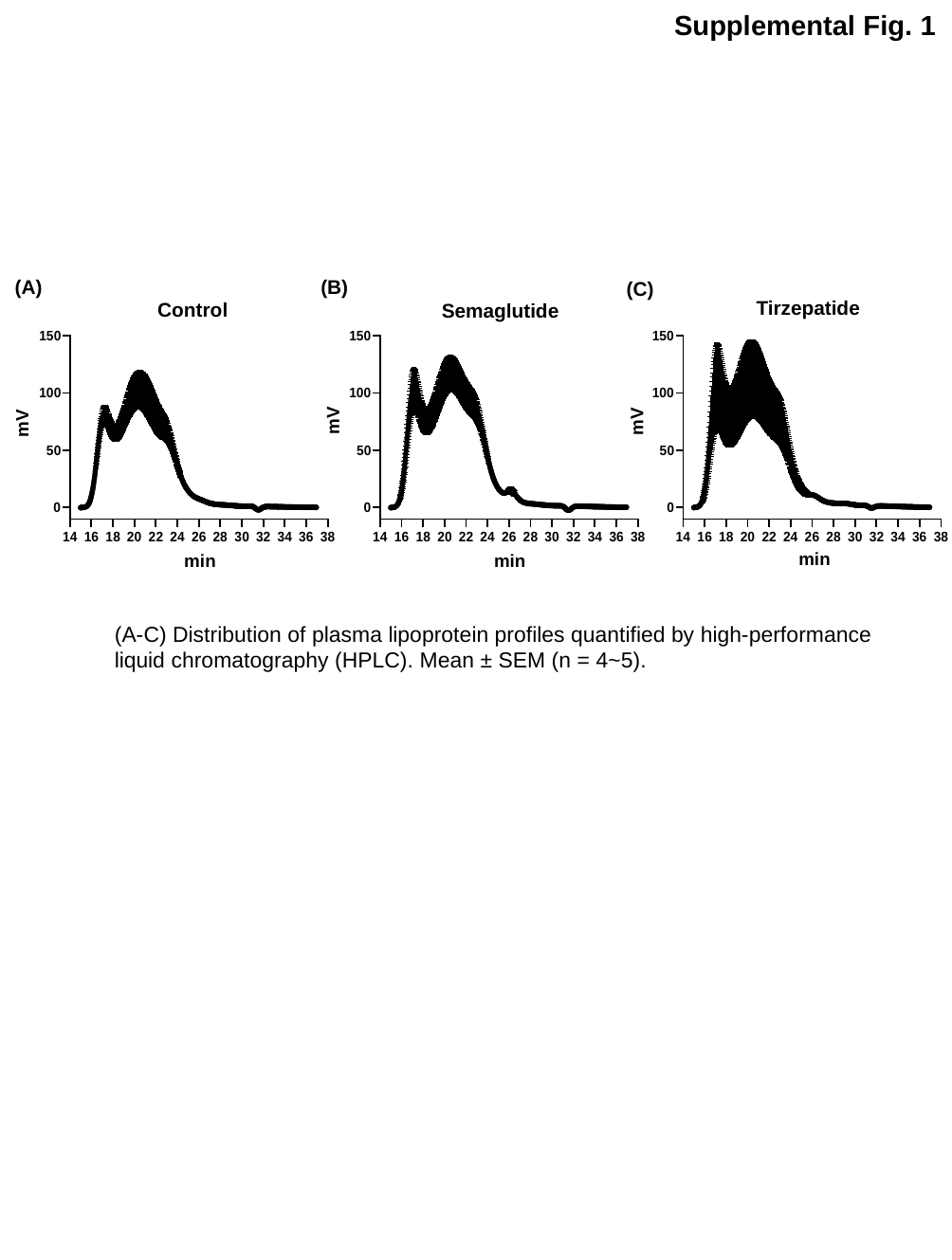

Supplemental Fig. 1
(A)
(B)
(C)
Tirzepatide
Control
Semaglutide
mV
mV
mV
min
min
min
(A-C) Distribution of plasma lipoprotein profiles quantified by high-performance liquid chromatography (HPLC). Mean ± SEM (n = 4~5).
