# Supplementary material for: Early intervention with tirzepatide or semaglutide influences anti-atherosclerotic effects in ApoE knockout mice
Source: Sci Rep. 2026 Apr 7;16:16718. doi: 10.1038/s41598-026-42437-8 (PMC13223216; doi:10.1038/s41598-026-42437-8)
Supplement: Supplementary file 2 — Supplementary Information 2. [file 41598_2026_42437_MOESM2_ESM.docx]

|  | Control | Semaglutide | Tirzepatide |
| --- | --- | --- | --- |
| T-chol (mg/dL) | 291.9 ± 49.4 | 353.6 ± 60.6 | 339.0 ± 100.0 |
| CM (mg/dL) | 79.4 ± 10.0 | 101.2 ± 22.2 | 102.1 ± 35.4 |
| VLDL (mg/dL) | 152.6 ± 31.9 | 174.9 ± 29.8 | 166.7 ± 49.4 |
| LDL (mg/dL) | 50.5 ± 7.7 | 62.7 ± 7.2 | 57.5 ± 15.1 |
| HDL (mg/dL) | 9.3 ± 0.7 | 14.8 ± 2.2 | 12.7 ± 2.6 |

Supplemental Table 1. Distribution of plasma lipoprotein fractions quantified by high-performance liquid chromatography (HPLC)

Mean ± SEM (n = 4~5). No significant differences detected by one-way ANOVA.
